# Validation of intracranial hemorrhage in the Norwegian Patient Registry

**DOI:** 10.1002/brb3.900

**Published:** 2018-01-23

**Authors:** Lise R. Øie, Mattis A. Madsbu, Charalampis Giannadakis, Anders Vorhaug, Heidi Jensberg, Øyvind Salvesen, Sasha Gulati

**Affiliations:** ^1^ Department of Neurology St Olavs Hospital Trondheim University Hospital Trondheim Norway; ^2^ Department of Neuroscience Norwegian University of Science and Technology (NTNU) Trondheim Norway; ^3^ Department of Neurosurgery St. Olavs Hospital Trondheim University Hospital Trondheim Norway; ^4^ The Norwegian Patient Registry Trondheim Norway; ^5^ Department of Public Health and General Practice Norwegian University of Science and Technology (NTNU) Trondheim Norway

**Keywords:** health registries, hemorrhagic stroke, intracranial hemorrhage, positive predictive value, subarachnoid hemorrhage, subdural hemorrhage, validation

## Abstract

**Objectives:**

Administrative health registries need to have accurate diagnoses and sufficient coverage in the population they serve in order to be useful in research. In this study, we investigated the proportion of discharge diagnoses of intracranial hemorrhage (ICH) that were coded correctly in the Norwegian Patient Registry (NPR).

**Materials and Methods:**

We reviewed the electronic medical records and diagnostic imaging of all admissions to St. Olavs University Hospital, Trondheim, Norway, between January 1, 2008, to December 31, 2014, with a discharge diagnosis of ICH in the NPR, and estimated positive predictive values (PPVs) for primary and secondary diagnoses. Separate calculations were made for inpatient and outpatient admissions.

**Results:**

In total, 1,419 patients with 1,458 discharge diagnoses of ICH were included in our study. Overall, 1,333 (91.4%) discharge diagnoses were coded correctly. For inpatient admissions, the PPVs for primary discharge codes were 96.9% for hemorrhagic stroke, 95.3% for subarachnoid hemorrhage, and 97.9% for subdural hemorrhage. The most common cause of incorrect diagnosis was previous stroke that should have been coded as rehabilitation or sequela after stroke. There were more false‐positive diagnoses among outpatient consultations and secondary diagnoses.

**Conclusion:**

Coding of ICH discharge diagnoses in the NPR is of high quality, showing that data from this registry can safely be used for medical research.

## 1 INTRODUCTION

Administrative and clinical health registries are established to monitor and manage healthcare services. Data from these registries can be used in epidemiological studies as they are easily obtained, include high number of patients, and provide both high external validity and statistical power (Jakola et al., 2012; Nerland et al., 2015; Salman et al., 2014; Sorensen, 1997). Administrative health registries need to have accurate diagnoses and sufficient coverage in the population they serve in order to be useful in research. Ischemic stroke accounts for approximately 80% of all strokes, and most studies investigating data quality of stroke in hospital registries focus on this entity. However, hemorrhagic stroke (HS) and subarachnoid hemorrhage (SAH) are associated with even higher morbidity, disability, and mortality rates, and further research on prevention, management, and outcomes of intracranial hemorrhage is warranted. The Norwegian Patient Registry (NPR) is a nationwide administrative health registry that can potentially provide useful data for research on intracranial hemorrhage (Gulati et al., 2015). The aim of this study was to investigate the proportion of ICH discharge diagnoses coded correctly in the NPR.

## 2 MATERIALS AND METHODS

### 2.1 Ethical approval

The regional committee for medical research in Central Norway approved this study (2014/958) and waivered the requirement of informed consent.

### 2.2 The Norwegian healthcare system

Norway, with its 4.7 million inhabitants (2009 Census, Statistics Norway), has geographically evenly distributed resources, and a public health care system for all inhabitants. Acute illness requiring hospital admission is treated free of cost by the public healthcare system, and insurance policies do not influence the diagnosis or treatment of ICH. Only public hospitals provide health care to patients with ICH, and the health authorities cover all inpatient treatment; thus, costs are generally not any concern for patients nor healthcare providers.

### 2.3 The Norwegian Patient Registry

NPR is a national administrative health registry containing person‐identifiable information on all inpatient and outpatient treatment by the public Norwegian specialist healthcare services. The database contains demographic, administrative, and health‐related data, such as dates of admission and discharge, the primary and unlimited number of secondary diagnoses according to the 10th revision of the International Classification of Diseases (ICD‐10), as well as codes for diagnostic and therapeutic procedures (Varmdal et al., 2016). All discharge diagnoses are exclusively assigned by the physician treating the patient and cannot later be altered. The registry receives data automatically on a monthly basis and is used for reimbursement to hospitals, hospital activity statistics, and research (Varmdal et al., 2016).

### 2.4 Study population

The study was carried out within the population of Sør‐Trøndelag County between January 1, 2008, and December 31, 2014. The study population of 298 000 individuals (2009 Census, Statistics Norway) is served by St. Olavs University Hospital, the only hospital in this well‐defined geographic region providing inpatient treatment of ICH. Patients with a primary or secondary diagnosis of ICH (ICD‐10 codes I60.0‐I62.0, I62.9, and S06.5) and a residential address within Sør‐Trøndelag County were included in the study (ICD‐10 code I60.0‐I62.0, I62.9, and S06.5). All included patients were aged 18 years or older.

### 2.5 Case ascertainment and validation

To verify the diagnosis of ICH, the electronic medical records of all the included patients were manually reviewed by four of the authors (LRØ, MM, HC, and AV). The review was based on all available information in the electronic medical records including diagnostic imaging, radiology reports, and laboratory tests.

Hemorrhagic stroke (HS) was defined as clinical symptoms of stroke combined with the presence of parenchymal hemorrhage on a cerebral CT (cCT) scan (ICD code I61.0‐I61.9). A diagnosis of subarachnoid hemorrhage (SAH) was made when typical clinical symptoms were present and at least one of the following findings: CT findings compatible with blood in the subarachnoid space, xanthochromic cerebrospinal fluid, or subarachnoid hemorrhage or aneurysm established on angiography (ICD‐10 code I60.0‐I60.9). Diagnosis of subdural hemorrhage (SDH) was also included in the study (ICD‐10 codes I62.0 and S06.5), defined as neurological symptoms and/or deficits combined with subdural blood on cCT scan. In clinical practice, the codes for traumatic SDH (S06.5) and nontraumatic SDH (I62.0) are used interchangeably. We accepted both codes as true‐positive cases if the diagnosis of SDH was confirmed.

Outpatient care was defined as medical service provided that did not require a hospital stay lasting longer than 5 hr, and inpatient care as medical care requiring hospitalization.

To detect possible cases of ICH not included in the registries, that is, false‐negative (FN) ICH, and to obtain an estimate of true‐negative (TN) ICH, we investigated 300 hospital admissions within the study period randomly selected by NPR, with main diagnoses that might clinically resemble or be caused by ICH. The size of the control group was based on the amount of patients permitted by NPR. Fifty cases in each of the following groups were investigated: (1) transient ischemic attack (G45); (2) stroke sequela (I69); (3) other specified cerebrovascular diseases (I67 and I68); (4) epilepsy (G40); (5) headache (G44); and (6) traumatic brain injury (S06).

Cases whose records were not found, or episodes of ICH occurring outside the study period, were excluded from the analyses. All incorrect cases (false positives) were reviewed again by the senior author (LRØ) who had expert knowledge of the inclusion criteria.

### 2.6 Statistics

Statistical analyses were performed with SPSS version 21.0. After reviewing the electronic medical records, we defined discharge codes from NPR as true positive (TP) or false positive (FP) and calculated the proportion of these codes (recorded diagnoses) that were cases of true ICH according to our predefined definitions (confirmed diagnoses). We defined this proportion as equivalent to positive predictive value (PPV). The PPV equals true‐positive cases divided by the sum of true‐positive and true‐negative cases (PPV = TP/[TP + TN]). We estimated PPV for primary and secondary diagnoses and made separate calculations for inpatient and outpatient admissions.

## 3 RESULTS

**Figure 1:**
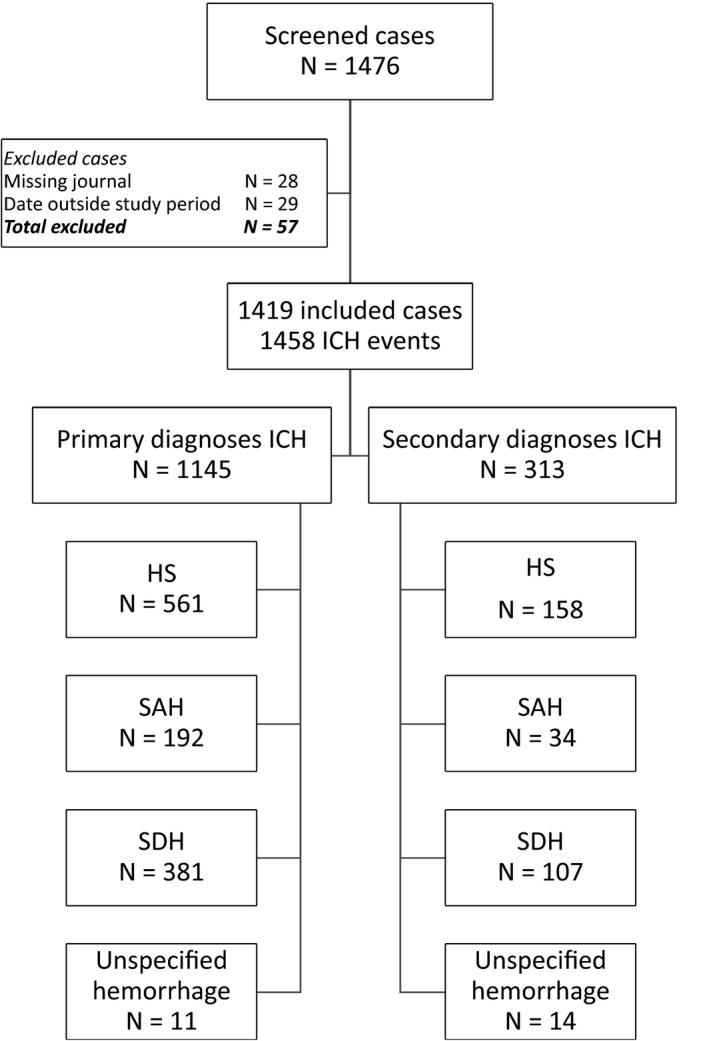
Study enrollment

Study enrollment is presented in Figure 1. In total, 1,476 cases of ICH were identified in NPR within the study period. We excluded 29 cases where the event occurred outside the study period and 28 cases with no available electronic medical records. Cases lacking electronic medical records were individuals with a residential address in Sør‐Trøndelag, but who received treatment at other hospitals in Norway or abroad. We did not have ethical approval to review medical records from other hospitals. A total of 1,419 cases were included in our analyses. The included cases had one primary or one/more secondary diagnosis of ICH. Altogether, the included cases had 1,458 discharge diagnoses of ICH; 1,145 primary diagnoses; and 313 secondary diagnoses of ICH.

Overall, 1,333 recorded discharge diagnoses were confirmed as ICH (91.4%). For inpatient admissions, HS as a primary diagnosis was confirmed in 528 of 545 cases (PPV 96.9%), SAH in 163 of 171 cases (PPV 95.3%), SDH in 334 of 341 cases (PPV 97.9%), and unspecified hemorrhagic stroke (ICD‐10 code I62.9) in 6 of 7 cases (PPV 85.7%) (Table 1). The PPVs of ICH as secondary diagnoses were 83.1% for HS, 55.9% for SAH, 96.0 for SDH, and 92.9% for unspecified HS (Tables 1 and 2).

**Table 1:** Positive predictive values, primary diagnoses

Recorded diagnoses from NPR	Confirmed diagnoses					
	Total	HS	SAH	SDH	Unspecified hemorrhage	Nonstroke events
Inhospital						
HS	545 (100)	528 (96.9)	1 (0.2)	3 (0.6)	—	13 (2.3)
SAH	171 (100)	3 (1.8)	163 (95.3)	—	—	5 (2.9)
SDH	341 (100)	2 (0.6)	1 (0.3)	334 (97.9)	—	4 (1.2)
Unspecified hemorrhage	7 (100)	—	—	—	6 (85.7)	1 (14.3)
Total	1064	533	165	337	6	23
Outpatient						
HS	16 (100)	7 (43.8)	—	—	—	9 (56.3)
SAH	21 (100)	—	—	—	—	21 (100)
SDH	40 (100)	—	—	34 (85.0)	—	6 (15.0)
Unspecified hemorrhage	4 (100)	—	—	—	1 (25.0)	3 (75.0)
Total	81	7	—	34	1	39

NPR, Norwegian Patient Registry; HS, hemorrhagic stroke; SAH, subarachnoid hemorrhage; SDH, subdural hemorrhage.

**Table 2:** Positive predictive values, secondary diagnoses

Recorded diagnoses from NPR	Confirmed diagnoses					
	Total	HS	SAH	SDH	Unspecified hemorrhage	Nonstroke events
Inhospital						
HS	154 (100)	128 (83.1)	—	2 (1.3)	—	24 (15.6)
SAH	34 (100)	—	19 (55.9)	—	—	15 (44.1)
SDH	101 (100)	—	—	97 (96.0)	—	4 (4.0)
Unspecified hemorrhage	14 (100)	—	—	—	13 (92.9)	1 (7.1)
Total	303	128	19	99	13	44
Outpatient						
HS	4 (100)	1 (25.0)	—	—	—	3 (75.0)
SAH	—	—	—	—	—	—
SDH	6 (100)	—	—	2 (33.3)	—	4 (66.7)
Unspecified hemorrhage	—	—	—	—	—	—
Total	10	1	—	2	—	7

NPR, Norwegian Patient Registry; HS, hemorrhagic stroke; SAH, subarachnoid hemorrhage; SDH, subdural hemorrhage.

In total, there were 125 (8.8%) FP diagnoses in 124 patients (Tables 3 and 4). There were more false‐positive cases among secondary discharge diagnoses and for outpatient admissions. For outpatient admissions, 13 cases coded as SAH in the NPR were cases of ingrown toenail, an example of a probable typing error, where the ICD‐10 code for ingrown toenail (L60.0) was incorrectly typed as SAH (I60.0). Compared to the other ICH subtypes, the PPV of SAH as a secondary diagnosis was lower. The most common cause of FP misclassification was cases of previous hemorrhagic stroke (38 out of 125 events) that should have been coded as rehabilitation (Z50.89) or sequela (I69.0‐I69.3) after stroke.

**Table 3:** Characteristics of false‐positive primary discharge diagnoses

Confirmed diagnoses										
Recorded diagnoses from NPR	Total	Other ICH subtype	Ischemic stroke	Previous stroke	TBI	Ingrown toe nail	Vascular myelopathy	Unruptured aneurysm	Unknown diagnosis	Other
Inhospital										
HS	17 (100)	4 (23.5)	1 (5.9)	1 (5.9)	8 (47.1)	—	1 (5.9)	—	—	2 (11.8)
SAH	8 (100)	3 (37.5)	1 (12.5)	—	—	—	—	4 (50.0)	—	—
SDH	7 (100)	3 (42.9)	1 (14.3)	—	3 (42.9)	—	—	—	—	—
Unspecified hemorrhage	1 (100)	—	—	—	—	—	—	—	—	1 (100)
Total	33	10	3	1	11	—	1	4	—	3
Outpatient										
HS	9 (100)	—	—	7 (77.8)	—	—	—	1 (11.1)	—	1 (11.1)
SAH	21 (100)	—	—	6 (28.6)	1 (4.8)	13 (61.9)	—	—	—	1 (4.8)
SDH	6 (100)	—	—	1 (16.7)	2 (33.3)	—	—	—	2 (33.3)	1 (16.7)
Unspecified hemorrhage	3 (100)	—	—	1 (33.3)	—	—	—	—	—	2 (66.7)
Total	39	—	—	15	3	13	—	1	2	5

NPR, Norwegian Patient Registry; HS, hemorrhagic stroke; SAH, subarachnoid hemorrhage; SDH, subdural hemorrhage; TBI, traumatic brain injury.

**Table 4:** Characteristics of false‐positive secondary discharge diagnoses

Confirmed diagnoses										
Recorded diagnoses	Total	Other ICH subtype	Ischemic stroke	Previous stroke	TBI	Ingrown toenail	Vascular myelopathy	Unruptured aneurysm	Unknown diagnosis	Other
Inhospital										
HS	26 (100)	2 (7.7)	3 (11.5)	12 (46.2)	6 (23.1)	—	—	—	—	3 (11.5)
SAH	15 (100)	—	1 (6.7)	5 (33.3)	1 (6.7)	—	1 (6.7)	3 (20.0)	3 (20.0)	1 (6.7)
SDH	4 (100)	—	—	1 (25.0)	1 (25.0)	—	—	—	2 (50.0)	—
Unspecified hemorrhage	1 (100)	—	—	—	—	—	—	—	1 (100)	—
Total	46	2	4	18	8	—	1	3	6	4
Outpatient										
HS	3 (100)	—	—	2 (66.7)	—	—	—	—	1 (33.3)	—
SAH	—	—	—	—	—	—	—	—	—	—
SDH	4 (100)	—	—	2 (50.0)	—	—	—	—	1 (25.0)	1 (25.0)
Unspecified hemorrhage	—	—	—	—	—	—	—	—	—	—
Total	7	—	—	4	—	—	—	—	2	1

NPR, Norwegian Patient Registry; HS, hemorrhagic stroke; SAH, subarachnoid hemorrhage; SDH, subdural hemorrhage; TBI, traumatic brain injury.

In the control group, we excluded 21 cases with no electronic medical record, leaving 279 cases included in our analysis. There were no false‐negative (FN) cases in the control group.

## 4 DISCUSSION

This study shows that coding of inpatient treatment of ICH in NPR is of high quality, and that data from this registry can be safely used for medical research. For hospital admissions registered in NPR with primary diagnoses of HS, SAH, or SDH, the proportion of cases coded correctly exceeded 95%. An exception was unspecified HS, with a PPV of 85.7%. The proportion of ICH coded correctly was higher for primary diagnoses and for inpatient admissions.

Interestingly, we did not detect any false‐negative cases of ICH in the control groups. The most common cause of false‐positive misclassification was previous ICH that should have been coded as sequelae or rehabilitation after stroke. These misclassifications were more common among secondary diagnoses and outpatient admissions. In an effort to identify new ICH only, and not cases of previous ICH, the addition of discharge codes of rehabilitation and sequela (ICD‐10 codes Z50.89 or I69, respectively) might be helpful. Outpatient diagnoses should not be included in future studies without review of medical records and diagnostic imaging.

The PPV in this study is higher than previously reported, showing that not all administrative registries and time periods are suited for research on ICH. A validation study of acute stroke in NPR during the time period of 1994 to 1996 found a PPV of 68% for acute stroke (ICD‐9 codes 430, 431, 434, and 436) (Ellekjaer, Holmen, Kruger, & Terent, 1999). A study from a Danish patient registry during the same time period estimated a PPV of 48% for SAH and 66% for HS (Johnsen, Overvad, Sorensen, Tjonneland, & Husted, 2002). Moreover, a study from Finland reported PPV of 86% for fatal and nonfatal strokes combined, with higher PPV for SAH and HS compared with ischemic stroke (Tolonen et al., 2007). More in line with our results, a US validation study from Washington State based on the Comprehensive Hospital Abstract Reporting System between 1990 and 1996 reported a PPV of 94% for SAH and 89% for HS (Tirschwell & Longstreth, 2002). A more recent study from the Czech Republic validating stroke diagnoses in a hospital discharge registry from 10 randomly selected hospitals showed a PPV of 91% for HS and 91% for SAH (Sedova et al., 2015). All the above studies validated coding of stroke diagnoses in general, and the numbers of included cases with ICH were lower than in our study. A study validating coding of strictly ICH between 2000 and 2008 in a UK database (The Health Improvement Network) showed a PPV of 91% for SAH and 73% for HS (Gaist, Wallander, Gonzalez‐Perez, & Garcia‐Rodriguez, 2013).

Studies validating diagnoses of stroke in health registries show higher PPV for ICH than for ischemic stroke. The use of cCT, CSF analysis, and clinical manifestations makes diagnoses of ICH more straightforward than for ischemic stroke and may be an explanation for this difference. All cases included in our study were treated at St. Olavs University Hospital with access to both a stroke unit and neurosurgeons, which may be another contributing factor to the high PPV. Tolonen et al. (2007) found that stroke events treated at university hospitals had the highest PPV for stroke diagnoses. Most hospitals in Norway have the equipment and diagnostic capabilities to establish a diagnosis of ICH, and we do not expect any major differences in coding practices of ICH between university and local hospitals.

Big data from clinical registries and administrative databases are useful for evaluating treatment strategies and add a different dimension to the results of more selective randomized trials. This kind of research is especially important in the field of neurosurgery, where large practice variations exist even for common conditions. In a retrospective population‐based parallel cohort study of patients with low‐grade gliomas, Jakola et al. (2012) were able to show that early surgical resection was associated with better overall survival than initial biopsy and watchful waiting. Another population‐based study from Scotland on patients with unruptured brain arteriovenous malformations showed that conservative management was associated with better clinical outcomes than treatment interventions (Salman et al., 2014). Using prospectively collected data from the Norwegian Registry for Spine Surgery, Nerland and coauthors were able to demonstrate that the effectiveness of laminectomy and microdecompression was equivalent in the management of lumbar spinal stenosis (Nerland et al., 2015). Another important aspect is that clinical registries and administrative databases allow inclusion of large patient groups that are ineligible for inclusion in randomized trials due to age and comorbidity (Giannadakis et al., 2016; Madsbu, Solberg, Salvesen, Nygaard, & Gulati, 2017). Moreover, research based on administrative databases allows monitoring of trends, costs, and complications of surgical procedures in a real‐world setting exemplified by Deyo et al. (2010) for lumbar spinal stenosis. Administrative databases are also a potential gold mine for performing pharmacoepidemiological research to investigate risks of intracranial hemorrhage in patients on medications with antithrombotic properties (Gaist et al., 2017; Gulati et al., 2015; Shin et al., 2015).

### 4.1 Study strengths and limitations

The main strength of our study is the population‐based setting comprising the total population of Sør‐Trøndelag County. Detailed information was retrieved from the electronic medical records, and we were able to validate discharge coding of the different subtypes of ICH. A possible limitation is the Norwegian reimbursement system that may influence coding practice and inspire excessive coding. This may be a contributing factor for the relatively large number of FP cases of HS found among the secondary diagnoses. Another limitation was that electronic medical records could not be retrieved for 28 patients. The most likely explanation is that these patients were treated at hospitals outside the geographic catchment area of our study. Still, the number of missing patients is relatively low and unlikely to influence our results. Sensitivity and specificity are frequently used estimates in studies validating data quality. These values are highly dependent on the sample size, how extensive the registration is, and how many cases are registered with low or even no probability of being ICH events. Sensitivity and specificity were therefore considered not to be meaningful measures of validity in this study.

## 5 CONCLUSION

This study shows that coding of inpatient treatment of ICH in NPR is of high quality and that this registry can provide valuable data in medical research. Efforts to further improve the validity in administrative registries can allow them to serve as even better data sources for research.

## CONFLICT OF INTERESTS

All authors declare that they have no conflict of interests.
